# Home Management of COVID-19 Patients: A Successful Model in Non-severe COVID-19 Patients in the Developing World

**DOI:** 10.7759/cureus.21605

**Published:** 2022-01-25

**Authors:** Syed Alishan, Farheen Ali, Zafar Iqbal, Ali Ammar, Atif S Muhammad, Faiza Farooq, Ayaz Mir, Nawal Salahuddin, Tahir Saghir, Musa Karim

**Affiliations:** 1 Adult Cardiology, National Institute of Cardiovascular Diseases (NICVD), Karachi, PAK; 2 Infectious Disease, National Institute of Cardiovascular Diseases (NICVD), Karachi, PAK; 3 Cardiology, National Institute of Cardiovascular Diseases (NICVD), Karachi, PAK; 4 Interventional Cardiology, National Institute of Cardiovascular Diseases (NICVD), Karachi, PAK; 5 Adult Critical Care Medicine, National Institute of Cardiovascular Diseases (NICVD), Karachi, PAK; 6 Adult Critical Care Medicine, King Faisal Specialist Hospital & Research Centre, Riyadh, SAU; 7 Statistics, National Institute of Cardiovascular Diseases (NICVD), Karachi, PAK

**Keywords:** risk assessment, home management, treatment, pandemic, covid-19

## Abstract

Background

Around 80-85% of coronavirus disease 2019 (COVID-19) cases were reported to have mild disease and home treatment of such patients was proved to be effective without significant morbidity or mortality. Therefore, the aim of this study was to assess the outcome of home management of non-severe COVID-19 infection in healthcare providers in the developing world.

Methods

This observational cohort study was conducted at the National Institute of Cardiovascular Diseases from June 2020 till January 2021. It included health care workers who tested positive for COVID-19 with non-severe infection and received home treatment. The COVID-19 management team monitored their symptoms and oxygen saturation over the phone. Need-based lab tests, X-rays, home proning, steroids, and oxygen were administered along with the standard intuitional management strategies. Study outcomes included duration of recovery, need for hospitalization, and expiry.

Results

A total of 128 patients were included, out of which 98 (76.6%) were male, and the mean age was 32.9 ± 5.9 years. Fever was the most common symptom, seen in 89.8% of patients. Most of the patients (85.9%) had no pre-existing comorbidities. Five patients received home oxygen therapy, seven received steroid therapy, and one received home pruning. The average time of recovery was 13.8 ± 8.1 days with no mortality; however, 14 (10.9%) patients were hospitalized due to worsening of symptoms.

Conclusion

Home treatment for COVID-19 patients with mild to moderate disease after appropriate risk assessment can be a safe and effective option to preserve hospital capacities for more needy and severely ill patients.

## Introduction

Coronavirus disease 2019 (COVID-19) became a global pandemic with its overwhelmingly high transmission rates after its outbreak in Wuhan in late 2019 [1]. Its human-to-human transmission occurs via droplets produced from the coughing and sneezing of infected persons. To prevent its spread, the World Health Organization (WHO) not only recommended the enforcement of extensive measures like mass-level lockdowns but also raised the alarm and recommended strict standard operating procedures (SOPs) for healthcare systems around the globe [1]. Subsequently, socioeconomic impacts, a higher rate of disease propagation, and anticipated significant morbidity or mortality made the fight against COVID-19 challenging, especially for less equipped healthcare systems of developing countries like Pakistan [2].

Since the start of the disease from 2019 till June 2021, more than 175,676,457 people were infected with COVID-19, and out of them, more than 3,790,320 people lost their lives [3]. For better management, WHO divides patients according to disease severity into three groups, with different proportions of patients in each group: critical (6.1%), severe (13.8%), and non-severe (80%) [4]. Initially, all patients were admitted to hospitals, which placed an increase huge burden on the health care system and subsequently reduced care and admissions for more deserving sick patients. On the other side, the fatality rate of hospitalized patients was reported to be 8-28%, which increased with the advancement of age [5]. Another big challenge was appropriate recommendations for the best treatment options for these patients because of the lack of trials and being a new disease [6]. In this scenario, an increasing number of people were coming to health care setups with different manifestations of COVID-19 like respiratory illnesses, fever, coughing, and severe myalgia [7].

The good thing in this scenario was that around 80% of the patients were presenting with non-severe disease [4,8] but one never knows when their disease severity increases and they move into the severe category, which is usually masked by “happy hypoxia” [7]. This needs a close monitoring system for non-severe patients to assess the development of signs and symptoms as well as for a drop in their oxygen saturation. This system was necessary to reduce the fear and to support those frontline health care workers who were devoting their lives to the management of patients in this pandemic. This type of system was also necessary to reduce the unnecessary hospital burden of patients and to reduce the movement of patients out of home isolation for regular checkups. This also reduces the spread of infection. National Institute of Cardiovascular Diseases (NICVD) is a tertiary care hospital that was facing the same scenario; this was managed by developing a system of monitoring of its health care workers who were infected with COVID-19 and were suffering from non-severe COVID-19 infection at their home. This study was conducted to evaluate the effectiveness of this system. Its results can then be safely applied to the general public to further reduce the hospital load and to treat the maximum number of patients in case of overburdening of the health care system.

## Materials and methods

This was an observational cohort study conducted at the COVID-19 care unit of the National Institute of Cardiovascular Diseases (NICVD), Karachi, Pakistan. It was conducted from June 2020 till January 2021. It was started after approval from the ethical review committee of the NICVD (approval number: ERC-30/2020). All those doctors and paramedics who gave consent and had symptoms of fever, cough, or shortness of breath and were diagnosed as COVID by a polymerase chain reaction (PCR) test were included in the study. Those patients who were asymptomatic and tested positive because of exposure were also included. All the patients who were included belong to the non-severe COVID group. All those patients were excluded who have symptoms but PCR was negative, or have a severe or critical disease at presentation, or were not health care providers.

Patients were classified, managed, and selected for study by the COVID-19 management team of the hospital, which included selected doctors from the departments of infectious diseases, critical care, and cardiology. This team was created to deal with patients with suspected and confirmed COVID. On the basis of World Health Organization (WHO) criteria, COVID patients were classified into non-severe, severe, and critical groups by this team. In the non-severe COVID category, those patients were included who were either asymptomatic or have symptoms but maintained oxygen saturation above 94% at room air and had clear lung fields on X-ray chest. The diagnosis of COVID-19 was made on the basis of a positive COVID PCR test via a nasal swab. After initial evaluation by the above team, patients were discharged and were followed at home via phone calls. On phone calls, the COVID management team assessed symptoms, temperature spikes, and oxygen saturation by thermometers and oxygen saturation probes, respectively, for each patient. Until they maintained an oxygen saturation of more than 94%, they were kept on symptomatic therapy, and X-rays and labs were only done if the patients' saturation dropped to less than 94%.

Data collection was done on a structured questionnaire, which included demographic data (gender and age), reported symptoms (fever, cough, shortness of breath, desaturation, body aches (myalgia), and gastrointestinal (GI) symptoms), and preexisting conditions (hypertension, asthma/COPD, digestive disorders, diabetes, and smoking) of all the enrolled patients (see Appendices). Laboratory and imaging assessments, such as chest X-ray (CXR), C-reactive protein (CRP), complete blood count (CBC), absolute lymphocyte count, ferritin, and lactate dehydrogenase (LDH) levels, were obtained for patients where ever needed based on clinical assessment and dropping of oxygen saturation. Study outcomes included the duration of recovery, need for hospitalization, and expiry.

Data were collected on a structured proforma and analysis was performed using Statistical Package for the Social Sciences (SPSS) version 19 (IBM Corp., Armonk, NY). Categorical data were summarized as frequency and percentages and mean ± standard deviations were computed for quantitative data.

## Results

A total of 128 doctors and paramedical staff of the institution who tested positive for COVID-19 and received home management during the first and second waves of the COVID-19 outbreak were included. Out of 128, 98 (76.6%) patients were male, the age range was from 21 to 58 years, with a mean age of 32.9 ± 5.9 years. The mean duration of symptoms was 10.0 ± 6.7 days. The most commonly reported symptom was fever (89.8%), as shown in Table 1.

**Table 1:** Distribution of demographic characteristics, symptoms, and co-morbid conditions of study subjects COPD = chronic obstructive pulmonary disease

	Total
Total (N)	128
Gender distribution	
Male	76.6% (98)
Female	23.4% (30)
Age (years)	32.9 ± 5.9
Symptoms	
Fever	89.8% (115)
Body aches	82.8% (106)
Cough	78.9% (101)
Shortness of breath	35.9% (46)
Desaturation	13.3% (17)
Gastrointestinal symptoms	32.8% (42)
Asymptomatic	1.6% (2)
Co-morbid conditions	
None	85.9% (110)
Asthma/COPD	7% (9)
Hypertension	4.7% (6)
Diabetes	3.1% (4)
Smoking	2.3% (3)

Oxygen saturation monitoring was the main tool of home management. It remained >94% in 108 (80.5%) of patients. No further hematological testing was done in these patients who were managed symptomatically. In the rest of the cases, lab investigations were conducted; the results are shown in Table 2.

**Table 2:** Results of laboratory investigations of COVID-19 patients managed at home

	Total
Total (N)	128
Oxygen saturation	
> 94%	80.5% (103)
≤ 94%	19.5% (25)
C-reactive protein	
Assessed	8.6% (11)
Mean ± standard deviation	2.5 ± 2.9
Normal	100% (11)
Abnormal	0% (0)
Complete blood count	
Assessed	10.9% (14)
Normal	100% (14)
Abnormal	0% (0)
Lymphocyte count	
Assessed	12.5% (16)
Normal	87.5% (14)
Abnormal	12.5% (2)
Ferritin level	
Assessed	4.7% (6)
Normal	83.3% (5)
Abnormal	16.7% (1)
Lactate dehydrogenase (LDH)	
Assessed	2.3% (3)
Normal	66.7% (2)
Abnormal	33.3% (1)

Among those patients who dropped their oxygen saturation, five patients (3.9%) received home oxygen therapy, seven patients (5.4%) received steroid therapy, and one patient received home proning. The average time of recovery was 13.8 ± 8.1 days; however, 14 patients (10.9%) were hospitalized due to worsening of symptoms. Of these re-hospitalized patients, chest X-ray was normal in nine patients, bilateral infiltrates were observed in two patients, and mild bilateral perihilar infiltrates, bilateral haziness, and mild right-sided pleural effusion were reported for one patient each, both of whom were managed at home or moved to hospital with no mortality in either case.

## Discussion

The COVID-19 outbreak proved to be the ultimate test of healthcare systems around the world, especially in resource-limited healthcare systems of low- and middle-income countries like Pakistan with weak governance, insufficient health policies, improper basic health facilities, and poor response of the general public towards protective measures and SOPs [2]. Under these circumstances, the healthcare system responded with multiple options to make better use of available resources; at-home management with close monitoring was one of them. Our experience of home management of low-risk patients shows promising results with no reported mortality and only a 10.9% need of hospitalization rate. A study conducted by Xu H et al. [9] reported the requirement of hospitalization in six out of 74 (8.1%) home-quarantined COVID-19 patients. It has been reported that if the environment is conducive enough to ensure safe management, up to 80% of the patients with mild disease can be managed in the community itself. In this management system, oxygen saturation checking is the main monitoring tool for these patients via pulse oximetry [10-11]. The safety of such a strategy depends on adequate counseling and proper monitoring. It is not only convenient for the patient but also we would be able to preserve hospital capacity for needier and severely ill patients [12].

Appropriate risk assessment of the patients is crucial. Patients with severe characteristics such as room air oxygen saturation of <90% and hypotension requiring inotropic support should be considered for intensive care admission. Patients with moderate characteristics, such as a room air oxygen saturation of <95%, heart rate of >110 bpm, respiratory rate of <24/min, fatigue, and systolic blood pressure >90 mmHg should be considered for in-hospital management, and patients with mild symptoms can be considered for home treatment for symptoms, such as fever, sore throat, or cough while maintaining adequate nutrition and hydration [13-15].

Patients included in this study were all healthcare professionals, presumably with a better understanding of disease progression, however, to adopt such a strategy for the general population, more comprehensive telemedicine infrastructure would be needed. The effectiveness of such initiatives during the current pandemic has been witnessed in various parts of the world. Zhai Y et al. reported substantial benefits of the Telemedicine Consultation System (ETCS) introduced by the National Telemedicine Center of China (NTCC) in remote patient monitoring, consultations, multidisciplinary care, prevention, spreading awareness education, and training [16]. Crane SJ et al. shared another success story of telemedicine at the Mayo Clinic; this virtual visit model was well accepted by the prospective clients for timely and safe health care during this pandemic [17]. The current pandemic has highlighted the importance of telemedicine infrastructure; with the learnings of the current pandemic, futuristic telemedicine infrastructure should be considered not an optional but a mandatory entity of the healthcare system [18-19].

The limitation of this study was the small sample size along with the inclusion of only health care workers of a single center. But this gave confidence that this system can be implemented on a larger scale in the society to offload non-severe COVID patients’ burden on the health care system of developing countries. The cost of a pulse oximeter is far less than the cost of hospitalization, so it is the most effective tool in the home management of these patients. This also highlights the need for an integrated futuristic telemedicine infrastructure.

## Conclusions

The home treatment option of COVID-19 patients with a mild disease by implementing proper monitoring is safe and effective. Our experience of the home management of patients with close monitoring showed promising results with no mortality reports and a low hospitalization rate. Monitoring oxygen saturation via a pulse oximeter is the best tool in the home monitoring model to detect a deterioration in the condition before it worsens. While in a healthcare system with limited resources, a home treatment strategy after appropriate risk assessment can help make better use of available resources and preserve hospital capacity for needier and severely ill patients.

## Figures and Tables

**Figure 1:**
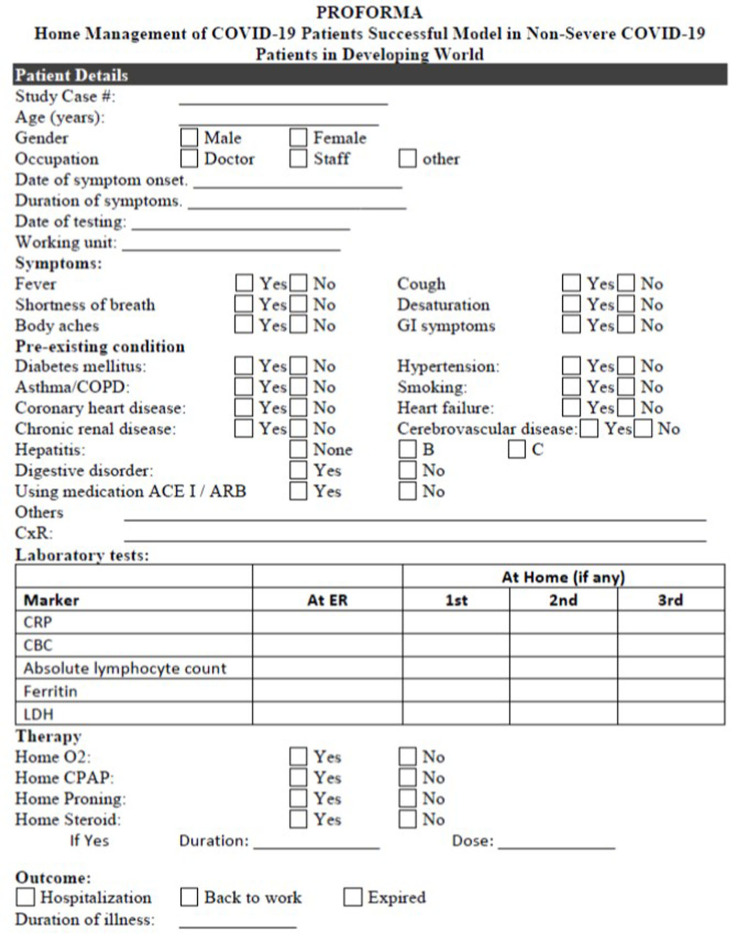
Study questionnaire
